# Analyzing the Quality and Challenges of Uncertainty Estimations for Brain Tumor Segmentation

**DOI:** 10.3389/fnins.2020.00282

**Published:** 2020-04-08

**Authors:** Alain Jungo, Fabian Balsiger, Mauricio Reyes

**Affiliations:** ^1^Insel Data Science Center, Inselspital, Bern University Hospital, University of Bern, Bern, Switzerland; ^2^ARTORG Center, University of Bern, Bern, Switzerland

**Keywords:** segmentation, brain tumor, uncertainty estimation, quality, deep learning

## Abstract

Automatic segmentation of brain tumors has the potential to enable volumetric measures and high-throughput analysis in the clinical setting. Reaching this potential seems almost achieved, considering the steady increase in segmentation accuracy. However, despite segmentation accuracy, the current methods still do not meet the robustness levels required for patient-centered clinical use. In this regard, uncertainty estimates are a promising direction to improve the robustness of automated segmentation systems. Different uncertainty estimation methods have been proposed, but little is known about their usefulness and limitations for brain tumor segmentation. In this study, we present an analysis of the most commonly used uncertainty estimation methods in regards to benefits and challenges for brain tumor segmentation. We evaluated their quality in terms of calibration, segmentation error localization, and segmentation failure detection. Our results show that the uncertainty methods are typically well-calibrated when evaluated at the dataset level. Evaluated at the subject level, we found notable miscalibrations and limited segmentation error localization (e.g., for correcting segmentations), which hinder the direct use of the voxel-wise uncertainties. Nevertheless, voxel-wise uncertainty showed value to detect failed segmentations when uncertainty estimates are aggregated at the subject level. Therefore, we suggest a careful usage of voxel-wise uncertainty measures and highlight the importance of developing solutions that address the subject-level requirements on calibration and segmentation error localization.

## 1. Introduction

Automated segmentation holds promise to improve the treatment of brain tumors by providing more reliable volumetric measures for treatment response assessment (Reuter et al., 2014) or by establishing new possibilities for high-throughput analysis, such as radiomics (Gillies et al., 2015). Over the past years, the improvements in automated brain tumor segmentation methods led to a steady increase in performance. This increase has two main reasons. First, the amount of annotated data has increased, leading to larger and more diverse datasets. Second, the available segmentation methods have evolved rapidly, especially with deep neural networks, which can leverage vast amounts of data. Although the results are reported to be close or on par with human performance (Meier et al., 2016; Bakas et al., 2018), there are still concerns about the clinical acceptability due to lower levels of robustness when compared to humans (Bakas et al., 2018). Possible reasons of this lack of robustness comprise the large variability of the imaging properties (e.g., different vendors, magnetic field strength, artifacts), and the intrinsic heterogeneity of brain tumors itself.

One promising direction to alleviate the problem of robustness is using uncertainty estimates of automated segmentation results. In segmentation, where a class label is assigned to each voxel, the uncertainty typically reflects the confidence level of the predicted class label. In that sense, uncertainty estimates provide additional information on a method's prediction and might be employed in various ways, e.g., as visual feedback, to guide or automate corrections via segmentation error localization, or for segmentation failure detection at the patient level (i.e., systems outputting a single estimate reflecting the quality of the automated segmentation). Methods producing uncertainty estimates for neural networks exist for over 20 years (MacKay, 1992; Neal, 1995) and evolved steadily (Blundell et al., 2015; Hernández-Lobato and Adams, 2015) but have only recently been adapted for large and complex deep models, such as those employed for brain tumor segmentation. The most popular methods are: (a) Monte-Carlo (MC) dropout proposed by Gal and Ghahramani (2016), (b) aleatoric uncertainty estimation introduced by Kendall and Gal (2017), and (c) uncertainty from ensembles as shown by Lakshminarayanan et al. (2017). Their popularity is mainly due to their ability to be used with state-of-the-art segmentation methods, requiring only minor modifications to architecture and training.

The additional information provided through the uncertainty estimates might be employed to quantify the segmentation performance or as a post-processing step to correct automatic segmentations. Being able to reliably quantify the segmentation performance is crucial when using uncertainty estimates in clinical applications. Roy et al. (2019) and Wang et al. (2019) quantified the segmentation performance at structure level by using structure-wise uncertainty estimates as a proxy to predict the Dice coefficient of automated segmentation results. Similarly, Eaton-Rosen et al. (2018) obtained improved calibration accuracy and more reliable confidence intervals of brain tumor volume estimates from structure-wise uncertainty. The segmentation quality can also be assessed at subject level, which is of interest in clinical applications to flag possible failure cases for expert review. For brain tumor cavity segmentation (Jungo et al., 2018b) did so by aggregating voxel-wise uncertainty. In skin lesion segmentation (DeVries and Taylor, 2018) proposed to train a separate model predicting the segmentation's Dice coefficient based on the input image, the automated segmentation result, and the voxel-wise uncertainty estimates. Further, the uncertainty estimates can be used to correct automated segmentations. Nair et al. (2018) and Graham et al. (2019) showed improved results by using uncertainty estimates to exclude highly uncertain multiple sclerosis lesions and glands, respectively. Both works exclude structures based on uncertainty and thus use task-related knowledge (e.g., multi-lesion segmentation). Directly correcting voxel predictions based on uncertainty is not suggested since this requires to overrule the segmentation model that was optimized to perform the segmentation task. This is especially true when segmentation and uncertainty estimates are provided by the same model.

**Figure 1:**
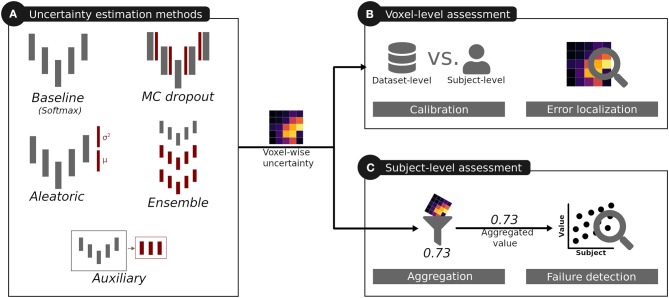
Overview of the analysis performed for the uncertainties produced by different uncertainty estimation methods. The red color indicates additions introduced by these methods with respect to the baseline.

Although uncertainty estimation methods have been applied to different segmentation tasks, little is known on their usefulness and limitations, nor a common evaluation of their quality has been reported for medical image segmentation. Therefore, we analyzed the most commonly used uncertainty estimation methods in regards to benefits and challenges for brain tumor segmentation, which is one promising clinical application for computer-assisted medical image segmentation. We considered the methods' calibration, their segmentation error localization, and their segmentation failure detection ability (see Figure 1 for an overview). This work builds on our previous work on the quality of uncertainties in medical image segmentation (Jungo and Reyes, 2019) and it is extended here in three aspects. First, based on our findings on observed deficiencies of voxel-wise uncertainty estimation approaches, we extend the work with experiments focusing on subject-level aggregation of uncertainty estimates. Second, to increase the clinical relevance of the analyses, we built and evaluated all methods for all three brain tumor labels (contrary to a simplified whole-tumor segmentation approach). Third, based on our previous work on the links between segmentation performance and quality of uncertainty estimates (Jungo et al., 2018a), we performed an experiment analyzing the effect of the training dataset size on the quality of uncertainty estimates.

## 2. Materials and Methods

### 2.1. Data

We used the BraTS 2018 training dataset (Menze et al., 2015; Bakas et al., 2017a,b,c, 2018) consisting of 285 subjects with high- and low-grade brain tumors. Each subject comprises images of the four standard brain tumor magnetic resonance (MR) sequences: T1-weighted (T1), T1-weighted post-contrast (T1c), T2-weighted (T2), and fluid-attenuated inversion recovery (FLAIR). Additionally, each subject holds a manual expert segmentation of three tumor sub-compartments: edema (ED), enhancing tumor (ET), and necrotic tissue combined with non-enhancing tumor (NCR/NET). In the official BraTS evaluation, these sub-compartments are combined into three hierarchical labels: whole tumor, tumor core, and enhancing tumor. Whole tumor (WT) is a combination of all tumor sub-compartments (i.e., ED, ET, NCR/NET), tumor core (TC) combines ET and NCR/NET, and enhancing tumor (ET) is defined by the ET sub-compartment. Aiming at yielding uncertainty estimates for these hierarchical tumor regions, we combined the tumor sub-compartment labels into the hierarchical labels before the training of the automated segmentation models.

The BraTS 2018 dataset comes pre-processed; the subjects and MR images are co-registered to the same anatomical template, resampled to unit voxel size (1 × 1 × 1 mm^3^), and skull-stripped. We additionally normalized each MR image subject-wise to zero mean and unit variance. For all our experiments, we subdivided the BraTS training dataset into a split of 100 training, 25 validation, and 160 testing subjects, stratified by the tumor grade.

### 2.2. Experimental Setup

We used U-Net-like (Ronneberger et al., 2015) architectures to asses uncertainty estimation methods. The reason for using U-Net-like architectures is twofold. First, the widely used U-Net-like architectures are still state-of-the-art in brain tumor segmentation (Isensee et al., 2018; Myronenko, 2018) and, second, their simplicity minimizes architectural influences in the uncertainty estimates. Inspired by Nikolov et al. (2018), our U-Net processes anisotropic subvolumes of five consecutive axial slices to predict the corresponding center slices. As in Nikolov et al. (2018), we adopted a full-slice view which motivated us to use 2D+1D convolutions (i.e., 2D in-plane convolution followed by 1D out-plane convolution) instead of using 3D convolutions. By considering only the valid part of the convolution, each 1D convolution in the encoder part thereby reduces the off-plane size by two, leading to a fully 2D decoder. The architecture consists of four pooling/upsampling steps with two convolutions for each encoder and decoder level. Every convolution is followed by dropout (*p* = 0.05) (Srivastava et al., 2014), batch normalization (Ioffe and Szegedy, 2015), and ReLU activation (Glorot et al., 2011). The architecture has four input channels corresponding to the four MR images (i.e., T1, T1c, T2, FLAIR) and three sigmoid outputs, one for each of the three tumor regions (i.e., WT, TC, ET). We note that a single softmax output that includes all labels is prohibited by the hierarchy of the tumor regions. A detailed description of the network architecture can be found in the Supplementary Section 1.1. Adaptations of the presented architecture to the individual uncertainty estimation methods are described in section 2.3).

We used a common training scheme for all uncertainty estimation methods. This scheme consists of training the network for 50 epochs, from where we selected the best performing models based on the mean Dice coefficient across labels on the validation set. Furthermore, we used Adam optimizer (Kingma and Ba, 2015) (learning rate: 10^−4^, β_1_: 0.9, β_2_: 0.999, ε: 10^−8^) to optimize the cross-entropy loss in mini-batches of 24. Extensive fine-tuning of the individual methods might introduce large differences in segmentation performance. Therefore, in order to minimize the influence of the segmentation performance on the uncertainty estimates, we purposely omitted extensive fine-tuning of the individual methods. Likewise, we did not perform any data augmentation to reduce possible influences on the uncertainty estimates.

### 2.3. Uncertainty Estimation Methods

For our experiments, we considered five methods (Figure 1A) producing voxel-wise uncertainty estimates: one baseline, three common methods, and one auxiliary approach. The three common methods were selected due to their popularity in medical image segmentation, stemming from their simple integration into state-of-the-art segmentation methods.

#### 2.3.1. Baseline: Softmax/Sigmoid Uncertainty

Although the softmax/sigmoid output is arguably a probability measure (Gal and Ghahramani, 2016), it is implicitly produced by classification networks. Therefore, we considered it as a reference comparison and named it *baseline*. We used the normalized entropy

(1) $$\begin{array}{l} H=-\left[p_{r}log\left(p_{r}\right)+\left(1-p_{r}\right)log\left(1-p_{r}\right)\right]\frac{1}{log\left(2\right)}\in \left[0,1\right] \end{array}$$

as a measure of uncertainty, with *p*_*r*_ being the sigmoid output of the network (see section 2.2) for tumor region *r*.

#### 2.3.2. MC Dropout

As shown by Gal and Ghahramani (2016), test-time dropout can be interpreted as an approximation of a Bayesian neural network. If applied during test time, dropout creates stochastic network samples that can be viewed as Monte-Carlo samples drawn from the posterior distribution of the network's weights. The foreground probability *p*_*r*_ of the tumor region *r* can be obtained by

$$p_{r}=\frac{1}{T}\overset{T}{\underset{t=1}{\sum }}p_{r,t},$$

where *T* is the number of samples. We used the normalized entropy (Equation 1) as a measure of uncertainty.

We considered four different dropout strategies. The first strategy consists of applying MC dropout throughout all layers (see presented architecture in section 2.2), whose minimal dropout (*p* = 0.05) was intended as regularization. The second strategy is inspired by existing work in segmentation uncertainty (Kendall et al., 2015; Nair et al., 2018), where dropout is applied only at key positions. Accordingly, we modified the architecture (see section 2.2) and applied a prominent dropout (*p* = 0.5) at the center positions of the U-Net architecture only, i.e., before the pooling and after the upsampling operations (cf. illustration of MC dropout in Figure 1A). The third strategy is similar to the second but introduces the center dropout (*p* = 0.5) only at the two lowest pooling/upsampling steps. In a fourth strategy, we replaced the dropout of the initial architecture by concrete dropout (Gal et al., 2017). Concrete dropout learns the dropout probability as part of the optimization procedure and can, as the standard dropout, also be applied during test time to generate stochastic network samples. We refer to this four strategies as *baseline+MC, center+MC, center low+MC*, and *concrete+MC*. We considered the non-MC counterparts *center, center low*, and *concrete* as additional softmax/sigmoid uncertainties next to *baseline* (described in section 2.3.1).

#### 2.3.3. Aleatoric Uncertainty

Aleatoric uncertainty is said to capture the noise inherent to an observation (Kendall and Gal, 2017) and is thus different from model uncertainty (e.g., MC dropout), which accounts for uncertainty in the model parameters. Kendall and Gal (2017) showed that aleatoric uncertainty in classification problems can be obtained by defining a network *f*(*x*) for input *x* that generates two outputs

$$\left[\hat{x},\sigma^{2}\right]=f\left(x\right),$$

where $\hat{x}$ correspond to the logits, and σ^2^ defines the variance of their Gaussian perturbation ($N\left(\hat{x},\sigma^{2}\right)$). The logits and the variance are simultaneously optimized by the aleatoric loss, which approximates the intractable objective with Monte-Carlo samples of the perturbed logits. We refer to this method as *aleatoric* and modified the architecture (see section 2.2) to output the variance $\sigma_{r}^{2}$ in addition to the logits ${\hat{x}}_{r}$ for every tumor region *r* (see Supplementary Section 1.1 for a detailed architecture description). We used the ${\hat{x}}_{r}$ outputs for the segmentations and the $\sigma_{r}^{2}$ outputs as measures of uncertainty. To normalize the range of the variance maps across tumor regions, we normalized it to [0, 1] over all subjects.

#### 2.3.4. Ensembles

Ensembles of neural networks are typically used when performance is highly relevant, e.g., for the BraTS challenge (Kamnitsas et al., 2017), but they can also be used to quantify uncertainties (Lakshminarayanan et al., 2017). Our ensemble consists of *K* = 10 models that share the same architecture (see section 2.2) but differ in training to enforce variability. We trained each model *k* on alternating *K* − 1 folds of the training dataset (as in k-fold cross-validation, resulting in 90 instead of 100 training subjects. We obtained the foreground probability *p*_*r*_ for each tumor region *r* by the average

$$p_{r}=\frac{1}{K}\overset{K}{\underset{k=1}{\sum }}p_{k,r}$$

of all models. As an uncertainty measure we used the normalized entropy (Equation 1).

#### 2.3.5. Auxiliary Networks

We use the term auxiliary network to describe additional networks that are trained successively to the primary network (i.e., segmentation network). Such networks have been used to assess segmentation performances by regressing subject-level performance metrics (DeVries and Taylor, 2018; Robinson et al., 2018). Inspired by this idea, we applied auxiliary networks for voxel-wise prediction of the segmentation errors (i.e., false positives, false negatives) of each tumor region separately. Since the auxiliary networks learn to detect segmentation errors, we can directly use their sigmoid segmentation error probabilities as a measure of segmentation uncertainty. Producing uncertainty estimates by a separate network is motivated by the presumption that a network might not be the best in assessing its own trustworthiness (Jiang et al., 2018).

We considered two types of auxiliary networks in our experiments. The first type, named *auxiliary feat*., uses the features maps of the segmentation network (see section 2.2) as input and consists of three consecutive 1 × 1 convolutions. The second type, named *auxiliary segm*., employs the three label maps (WT, TC, ET) produced by the segmentation network in combination with the four MR images as input. The difference between the two types of auxiliary networks is defined by the link to the segmentation network. The first is more closely linked through the feature maps, whereas the second is decoupled and only requires the resulting segmentations. We refer to the Supplementary Section 1.2 for a detailed description of the *auxiliary feat*. and *auxiliary segm*. architectures.

### 2.4. Analyzing Voxel-Wise Uncertainty

We selected three techniques to analyze the quality of voxel-wise uncertainties produced by the different uncertainty estimation methods (Figure 1B) independently of their expressed uncertainty (e.g., model uncertainty, data uncertainty). The techniques aim at evaluating the model's confidence levels and the segmentation error localization abilities, which are required for tasks relying on visual feedback, or guided/automated correction. Additionally, the Dice coefficient was used to monitor the segmentation performance.

#### 2.4.1. Reliability Diagram

Reliability diagrams (DeGroot and Fienberg, 1983) assess the quality of a model's confidence. It is a visual measure of how close a model's calibration is to practically unachievable perfect calibration (Guo et al., 2017). Perfect calibration is obtained when a model's predictions *f*(*x*) with confidence *p* are correct with a rate of *p* for any label *y*

P(y(x)=y|f(x)=p)=p,

where *y*(*x*) are the model's label predictions. For instance, when a model is confident with 70%, it should be correct 70 out of 100 times (Guo et al., 2017). To create a reliability diagram, the continuous predictions *f*(*x*) are discretized in *M* confidence bins *c*_*m*_ for *m* ∈ {1, …, *M*} and plotted against the accuracies *a*_*m*_ in these bins. Therefore, the identity line of the reliability diagram represents perfect calibration.

For segmentation tasks, the reliability diagrams are typically reported over an entire test set, jointly considering the confidences of all voxels across subjects. Although this offers a general idea of the model's overall calibration, it omits information about a single subject (i.e., patient). Achieving good calibration levels at subject-level is, however, required in a clinical setting if the voxel confidences should be used for visual feedback or guided corrections of automated segmentation results. Therefore, we report subject-level calibration along with dataset-level calibration.

Calibration builds on model confidence, which we used as a surrogate for uncertainty (as in Kendall and Gal, 2017). This consists in considering the tumor region probability *p*_*r*_ for the baseline, MC dropout and ensemble variants. Since aleatoric and auxiliary variants do not explicitly output probabilities *p*_*r*_, we translated their uncertainty by *y*(1 − 0.5*q*) + (1 − *y*)0.5*q* to confidence values, where *y* ∈ {0, 1} is the segmentation label and *q* ∈ [0, 1] is the normalized uncertainty.

#### 2.4.2. Expected Calibration Error

The expected calibration error (ECE) (Naeini et al., 2015) distills the information of a reliability diagram into one scalar value. It is defined by the absolute calibration error between the confidence and accuracy bins, *c*_*m*_ and *a*_*m*_, respectively, weighted by the number of samples *n*_*m*_ (in our case voxels) in the bin. More formally, with *N* and *M* being the total number of samples and the number of bins, the ECE is given by

$$ECE=\overset{M}{\underset{m}{\sum }}\frac{n_{m}}{N}|c_{m}-a_{m}|.$$

The ECE ranges from 0 to 1, where a lower value represents a better calibration. Through weighting by the bin size, the ECE is influenced by large confident and accurate extra-cranial regions typically found in brain tumor MR images. To reduce this effect, we only considered voxels within the skull-stripped brain to calculate the ECE. As for the reliability diagram, we are interested in the subject-level ECE and thus report the mean subject ECE instead of the dataset ECE (i.e., considering all voxels in the test set to calculate a single ECE). Complementary to the ECE, we also computed the average calibration error (Neumann et al., 2018). We refer to the Supplementary Section 4 for the description and results.

#### 2.4.3. Uncertainty-Error Overlap

In segmentation, not only calibration is of interest but also the model's ability to localize segmentation errors. Ideally, a model would be uncertain only where it makes mistakes. To assess this behavior, we introduce the uncertainty-error overlap (U-E). The U-E measures the overlap, through Dice coefficient, between the regions where the model is uncertain *U* about its prediction and the segmentation error *E* (i.e., union of false positives and false negatives), such that

$$U-E=\frac{2|U\cap E|}{\left|U|+|E\right|},$$

where |·| represents the cardinality. The U-E ranges from 0 to 1 with 1 describing a perfect overlap. By considering voxels belonging to *U* and *E* only, the U-E is not influenced by the true negative uncertainty and thus typically independent of the image size or additional background voxels, as opposed to the ECE. However, calculating U-E requires to threshold *U*. We determined the threshold for each method independently, based on the maximal U-E performance on the validation set. The U-E performance was evaluated for thresholds from 0.05 to 0.95 in steps of 0.05. Complementary to the U-E, we also computed the area under the curve of the precision-recall curve. We refer to the Supplementary Section 4 for the description and results.

#### 2.4.4. Dice Coefficient

Although the Dice coefficient is not a measure for analyzing the quality of the uncertainty, we used it to monitor the segmentation performance of the different methods. It measures the overlap between two segmentation and ranges from 0 to 1, where 1 describes perfect overlap. Rather than determining the best method for segmentation, the Dice coefficient monitoring aims at detecting potential influences of the segmentation performance on the uncertainty estimates. Ideally, all methods would produce identical segmentations attributing any improvement in uncertainty measures directly to the corresponding method. In practice, however, this is unfeasible due to differences in the architectures and training. An improvement in the uncertainty measures could, therefore, also be due to an improved segmentation performance.

### 2.5. Analyzing Aggregated Uncertainty at the Subject Level

Besides analyzing the method's uncertainty estimates on a voxel level, we further analyzed their quality when aggregated on a subject level (i.e., one scalar value per subject; Figure 1C). The motivation of the subject-level analysis is twofold. First, the aggregation distills the uncertainty information such that the influence of irrelevant and erroneous voxel-wise information is reduced. The aggregation, therefore, provides an assessment of the individual uncertainty estimations at a higher level that can forgive deficiencies (e.g., poor calibration) at the voxel level. Second, the aggregation presents a possible usage of the uncertainty estimations. It is an alternative to corrections at the voxel-level which are unfeasible for brain tumor segmentation where task-related knowledge (e.g., multiple lesions) is very sparse. In clinical applications subject-level information is important to flag possible failure cases for expert review. The vast amount of possible aggregations can further help in pointing to important characteristic of the voxel-wise uncertainty when used at the subject level. The quality of the aggregated subject-level information is defined by its relation to the segmentation performance; the better the aggregated uncertainty, the better it should be able to describe the segmentation performance. We aim at a good correlation between aggregated uncertainty and segmentation performance, which consequently enables accurate segmentation failure detection.

#### 2.5.1. Aggregation Methods

The aggregated subject-level scalar is highly influenced by the chosen aggregation method. Hence, we studied three distinct aggregation methods.

**Mean aggregation**. Mean aggregation is one of the simplest aggregation methods, and it is motivated by the intuition that an overall higher voxel-wise uncertainty should be an indicator of poor segmentation performance. This requires the aggregated, but not necessarily the voxel-wise, uncertainty to be calibrated. In practice, we used the negative mean uncertainty to obtain direct relation to the segmentation performance.

**Prior knowledge-based aggregation**. We know that uncertainty is inherently present at the segmentation boundary. Although this boundary uncertainty might be well-calibrated it is mainly proportional to the size of the segmentation and consequently introduces a bias toward the tumor size to the aggregation. Similarly, one might expect more severe issues when the large amount of uncertainties are present far from the segmentation boundary. If only boundary uncertainty is present we would expect less deviation from a reference segmentation. We used this knowledge to create three different aggregation weightings which deemphasize uncertainty at boundaries. The first weighting consists of masking out voxels at the segmentation boundary. In our experiments we masked three voxels within the boundary (i.e., one-pixel distance inside and outside, and at the boundary). The second weighting considers the distance to the boundary, penalizing uncertainties close to the boundary, and up-weighting uncertainties distant from the segmentation boundary. The third weighting normalizes the boundary uncertainty by dividing through the segmentation volume.

To aggregate the differently weighted voxel information to a scalar value per subject, we used three simple operations: mean, sum, and logsum (as used by Nair et al., 2018). We considered nine combinations between prior knowledge-based weightings and these three simple operations. The nine combinations were then used to train a random forest regressor that predicts the Dice coefficient of the segmentation. We used such a prediction model instead of evaluating the correlation with the segmentation performance because we aim at obtaining a good predictor rather than solely finding the most important combination. We refer to the Supplementary Sections 2.1, 2.3 for details regarding the nine combinations and training details of the random forest regressor.

**Aggregation with automatically-extracted features**. Instead of manually defining additional aggregation methods, we employed the PyRadiomics^1^ (Van Griethuysen et al., 2017, version 2.2.0) package to extract subject-level features from the voxel-wise uncertainty estimates automatically. Although typically used in the context of radiomics, the package is not limited to this application but is rather a general tool to extract shape, first-order, and other gray-level features. The benefit of using automated feature extraction is two-fold: (a) it allows us to compare to the aggregation with prior knowledge and (b) potentially points to new predictive features of the uncertainty. We extracted 102 features from the thresholded voxel-wise uncertainty estimates. The threshold was determined for each uncertainty method by the maximal U-E performance on the validation set (identical to section 2.4.3). The features were used to train a random forest regressor that predicts the Dice coefficient of the segmentations. We refer to the Supplementary Sections 2.2, 2.3 for features and training details.

#### 2.5.2. Subject-Level Metrics

We assessed the three aggregation methods for each uncertainty estimation method based on their ability to predict the Dice performance of the automated segmentations. To do so, we evaluated the estimates of the aggregation methods by three metrics.

**Spearman's rank correlation**. We used Spearman's rank correlation coefficient to asses the correlation between the estimated and the actual Dice coefficients. Spearman's rank correlation was chosen since not all estimates lead to a linear relationship (i.e., mean aggregation). The metric ranges from -1 to 1, where the extremes describe a perfect monotone relation (positive if 1, negative if −1) between estimated and actual Dice coefficients.

**AUC-ROC**. We evaluated the segmentation failure detection abilities of the uncertainty and aggregation methods by the area under the curve of the receiver operating characteristic (AUC-ROC). To do so, we translated the regression problem (i.e., building a predictor for the Dice coefficient) to a binary classification problem. We classified the segmentations in successful and failed according to the average inter-rater Dice coefficient for every tumor region. As the inter-rater performances are not provided for the BraTS 2018 dataset, we considered the inter-rater performances reported in Menze et al. (2015) for the BraTS 2013 dataset^2^. The AUC-ROC was computed by the scores of the regression output and ranges from 0 to 1, where 1 describes a perfect separator between the classes, 0.5 corresponds to random guesses, and 0 is the reciprocal of a prefect separator (i.e., consistently predicting wrong class).

**Youden's accuracy**. For improved comparability and understanding, we evaluated the accuracy (range [0, 1]) along with the AUC-ROC. We used the maximal Youden's index (Youden, 1950) to determine the accuracy from the ROC curve. This index is defined for each point on the ROC curve as

J=sensitivity-(1-specificity)

and corresponds to the vertical distance to the chance line (i.e., *sensitivity* = 1 − *specificity*). Its maximum defines an optimal point on the ROC curve.

## 3. Results

### 3.1. Dataset-Level vs. Subject-Level Calibration

**Figure 2:**
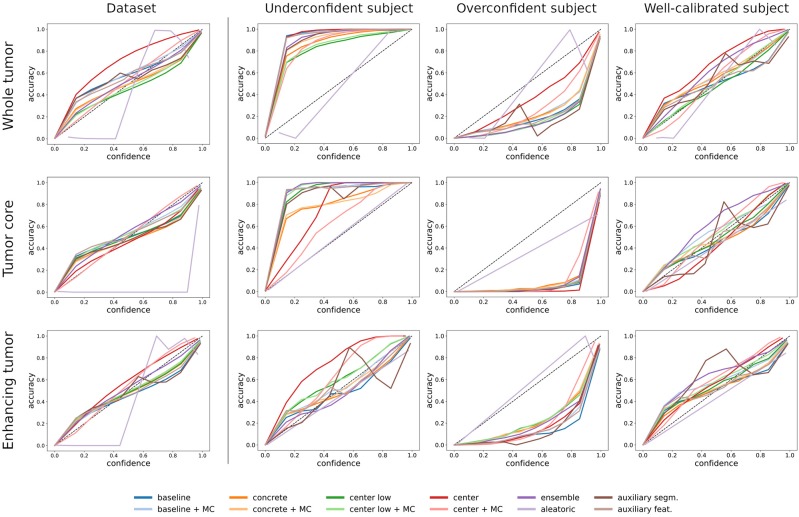
Comparison between dataset-level and subject-level calibration (shown in reliability diagrams) for the selected uncertainty estimation methods. The first column shows the dataset-level calibration, which considers all voxels in the dataset. The second to fourth columns show subject-level calibrations, which consider voxels of a single subject. The exemplary subjects indicate underconfident, overconfident, and well-calibrated methods. The rows indicate the three tumor regions.

Figure 2 illustrates the difference between dataset-level (i.e., all voxels in the test set) and subject-level (i.e., all voxels of a subject) calibration with reliability diagrams. While the calibration at the dataset level is good for all tumor regions, miscalibrations in the form of overconfidence and underconfidence are present at the subject level. We find an under-/overconfidence in 39%/25%, 30%/32%, and 21%/41% of the test subjects for the three tumor regions WT, TC, and ET. Consequently, less than 40% (36%, 38%, and 38%) of the subjects are well-calibrated. The percentages indicate that the amount of miscalibration is similar for all tumor regions, but ET exhibits more overconfidence (and less underconfidence) than the other regions (column *underconfident subject* in Figure 2 is exemplary). Also, we observe small differences among the uncertainty methods; they mostly agree, except for the aleatoric uncertainty, which disagrees at the dataset level.

### 3.2. Voxel-Wise Uncertainty

We evaluated the voxel-wise uncertainties on average subject-level ECE, uncertainty-error overlap (U-E), and Dice coefficient. The results are listed in Table 1 and reveal that no uncertainty estimation method considerably outperforms others. Most methods perform in a similar range with a small advantage for the *ensemble* method. Only the *aleatoric* method is distinctly performing worse in term of the uncertainty metrics ECE and U-E, while the competitive Dice coefficients indicate no segmentation related issues. We found that the MC dropout variants typically marginally outperform the non-MC variants (i.e., dropout only applied during training), but occasionally lead to considerable gains in ECE. The results also show that finding the optimal dropout strategy, i.e., the amount and position of dropout, is not evident. On one hand, the method containing moderate dropout (*center low/+MC*) outperforms the methods with minimal (*baseline/+MC*) and maximal (*center/+MC*) dropout on all metrics. On the other hand, the benefit of using MC dropout is larger for the minimal and maximal dropout strategies. Concrete dropout, which learns an optimal dropout rate, yielded comparable but not superior results than (*center low/+MC*). Furthermore, the results show that the auxiliary methods achieved uncertainty performances on par with the *baseline* model, on whose segmentation errors they are trained. Benefits in comparison to *baseline* are mainly found for *auxiliary feat*. and in terms of U-E.

**Table 1:** Performances of the different uncertainty estimation methods in terms of expected calibration error (ECE), uncertainty-error overlap (U-E), and Dice coefficient.

	**WT**			**TC**			**ET**		
	**ECE%**	**U-E**	**Dice**	**ECE%**	**U-E**	**Dice**	**ECE%**	**U-E**	**Dice**
Baseline	1.059	0.427	0.869	0.853	0.41	0.767	0.309	0.401	0.692
Concrete	0.984	0.429	0.875	0.802	0.419	0.775	0.278	0.407	0.686
Center low	0.942	0.434	0.88	0.83	0.409	0.775	0.28	0.403	0.686
Center	1.606	0.425	0.817	1.086	0.41	0.695	0.381	0.395	0.642
Baseline + MC	1.016	0.433	0.869	0.805	0.41	0.765	0.284	0.403	0.693
Concrete + MC	0.952	0.431	0.877	0.785	**0.422**	**0.778**	**0.27**	0.409	0.689
Center low + MC	0.922	0.435	**0.881**	0.83	0.41	0.769	0.275	0.409	0.69
Center + MC	1.014	0.432	0.874	1.06	0.409	0.716	0.462	0.4	0.651
Ensemble	**0.893**	**0.436**	0.88	**0.749**	0.402	**0.778**	0.275	0.411	**0.701**
Aleatoric	12.187	0.001	0.874	2.407	0	0.757	1.284	0.007	0.673
Auxiliary segm.	1.058	0.428	0.869	0.887	0.397	0.767	0.323	0.39	0.692
Auxiliary feat.	1.057	0.433	0.869	0.852	0.403	0.767	0.318	**0.423**	0.692

*All metrics range from 0 to 1, but the ECE is reported in % for better comparisons. Lower ECEs are better as well as higher U-Es and Dice coefficients.We note that the Dice coefficient is not a measure for analyzing the quality of the uncertainty and is reported to monitor the segmentation performance of the different methods. Mean values are presented, and standard deviations are omitted due to marginal differences. Bold values indicate best performances. Horizontal separations group types of uncertainty methods and WT, TC, and ET indicate the tumor regions whole tumor, tumor core, and enhancing tumor*.

Overall, the results are similar for all tumor regions. Differences among the tumor regions are mainly found in the ECE metric, which is considerably lower for ET than WT. This effect can be explained since the ET includes substantially fewer voxels predicted as uncertain (since fewer foreground voxels) and, in turn, the ET tumor class includes more certain background voxels, leading to an improved ECE. Furthermore, the results indicate a link between segmentation performance and ECE, where better-performing methods often relate to an improved ECE. Methods outputting the uncertainty estimates separately from the segmentation (i.e., *auxiliary segm., auxiliary feat*., and *aleatoric*) are excluded from this observation.

**Figure 3:**
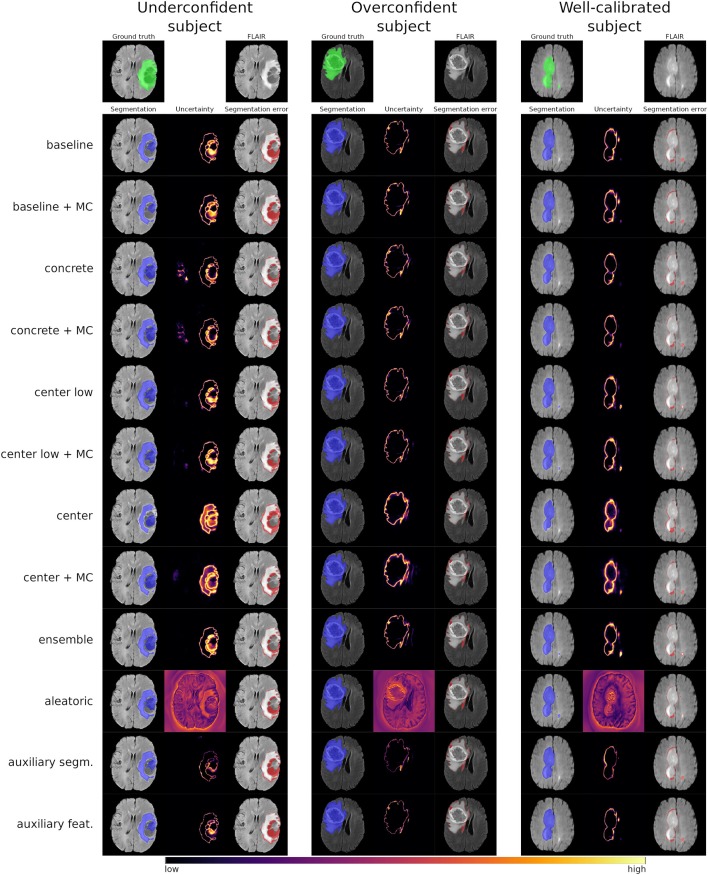
Visual examples of the whole tumor uncertainty produced by the different uncertainty estimation methods. The columns correspond to underconfident, overconfident, and well-calibrated subjects (same as in Figure 2).

Figure 3 shows the uncertainty estimates for the WT label (see Figures S3, S4 for visual examples of TC and ET) produced by the selected methods on underconfident, overconfident, and well-calibrated subjects (same subjects as in Figure 2). The examples visually confirm the similar segmentation performances of the different methods. Further, the uncertainty estimates clearly show a pattern between amount of uncertainty and miscalibration. The underconfident subject exhibits considerably more overall uncertainty than the overconfident subject and perceivably more than well-calibrated subject. We also observe that the amount of uncertainty varies among the methods. For instance, the *center/+MC* methods consistently exhibit more uncertainty than the auxiliary methods. The regions exhibiting uncertainty are, however, similar for all methods, except the *aleatoric* method which visually confirms its poor calibrations.

**Figure 4:**
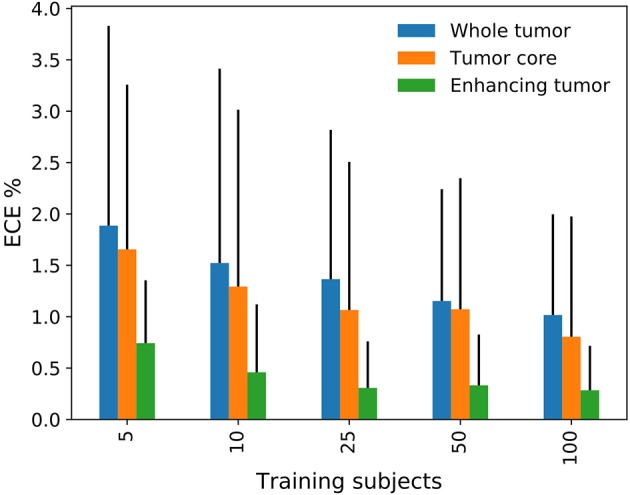
Effect of the training dataset size on the expected calibration error (ECE).

In an additional experiment, we analyzed the dependency of the training dataset size on the quality of uncertainty estimates. The method we used for these experiments is *baseline*+*MC* as it is mostly represents the performance level of the studied methods. The results in Figure 4 show that quality in terms of ECE is low (i.e., high ECE) with few training data and increases afterwards. This demonstrates that the higher uncertainty introduced through small datasets is worse in terms of quality.

### 3.3. Subject-Level Aggregated Uncertainty

**Figure 5:**
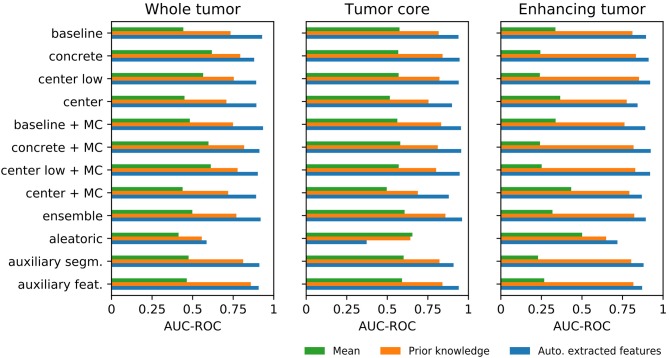
Differences among the three aggregation methods for each uncertainty estimation approach in terms of area under the curve of the receiver operating characteristic (AUC-ROC) for segmentation failure detection.

Figure 5 shows the AUC-ROC results of the uncertainty estimation methods for the three aggregation methods (see Figure S5 and Table S5 for the corresponding ROC curves and the AUC-ROC values, respectively). The results demonstrate that mean aggregation has a limited ability to detect segmentation failures and is comparable with guessing (i.e., AUC-ROC of 0.5). Results for the ET tumor label are even below 0.5, revealing a direct relation between uncertainty and segmentation performance instead of the expected inverse relation. An improvement over mean aggregation is achieved by aggregating with prior knowledge and automatically extracted features. The aggregation with automatically extracted features obtained the overall best AUC-ROC values. Although it is not possible to determine the best uncertainty method visually, the *aletoric* method shows apparent weaknesses. We also built a combined model with the automatically extracted features and the generated prior knowledge features, but it did not lead to consistent improvements in terms of AUC-ROC.

We assessed feature importance by accumulating their ranks over the individual regression models (i.e., one for each uncertainty estimation method). We found the *distance weighted masked mean* feature to be the most important feature for the prior knowledge-based aggregation. For the aggregation with automatically extracted features, the *shape sphericity*, which is a measure of roundness relative to a sphere, and *run length non-uniformity*, which is a measure of similarity among the different gray level run lengths, were dominantly the two most important features.

**Figure 6:**
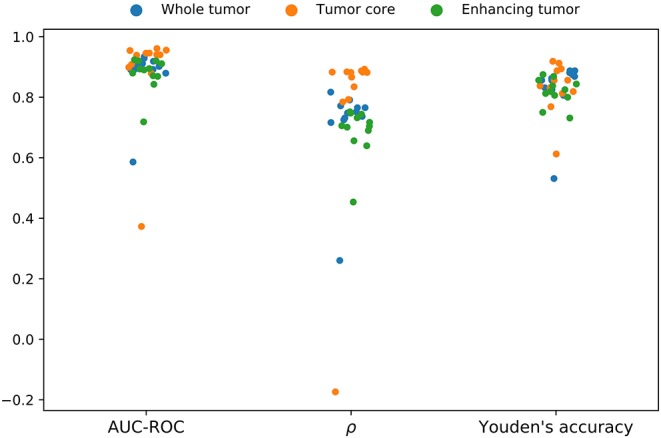
Segmentation failure detection performance of the aggregation uncertainty by automatically extracted features in terms area under the curve of the receiver operating characteristic (AUC-ROC) and Youden's accuracy as well as correlation with the segmentation performance in terms of Spearman's rank correlation (ρ). Each point per color represents an uncertainty estimation method. The negative outliers in each metric and for each tumor region correspond to the *aleatoric* method.

Since it represents the best-performing aggregation method, we evaluated the aggregation with automatically extracted features in terms of Spearman's rank correlation and Youden's accuracy in addition to AUC-ROC. The corresponding results are shown in Figure 6 with numerical details in Table S5. The results reconfirm the similarities found among the different uncertainty estimation methods, with the *aleatoric* method yielding the lowest performance, and producing negative outliers in all three metrics, although less prominent for the Youden's accuracy. Additionally, we observed that the predictions based on the TC tumor label uncertainty achieved the highest values for all three metrics, whereas ET tumor label uncertainty is typically the worst-performing.

## 4. Discussion

Uncertainty estimation methods have been used for different medical image segmentation tasks, but little is known on their quality and limitations. Therefore, we analyzed the quality of common uncertainty estimation methods on the clinically relevant problem of automated brain tumor segmentation. The methods were evaluated on their calibration, segmentation error localization, and segmentation failure detection abilities. First, our results show that overall good calibration is only achieved at the dataset level. Second, segmentation error localization relying on voxel-wise uncertainty is difficult and unreliable. However, we found that segmentation failure detection on subject level is possible by aggregating voxel-wise uncertainty estimates.

We found a good calibration of voxel-wise predictions at the dataset level but observed notable miscalibrations when assessed at the subject level. As such, the good dataset-level calibration can be explained by the subject-level miscalibrations (under- and overconfidence), which average out when combined. The subject-level miscalibrations are influenced by the dependence of neighboring voxels in the uncertainty estimates, resulting from the fully-convolutional architectures and an inter-voxel dependence in the MR images itself. Although this dependence is beneficial for the segmentation task, it also produces similar uncertainties within a neighborhood and therefore biases the calibration. Consequently, poor segmentations are expected to introduce a larger bias. The observed miscalibrations further indicate that the uncertainty estimates may contain non-negligible errors. As a consequence, using voxel-wise uncertainty for user feedback or guided corrections is questionable and might lead to undesired outcomes in automated corrections. Therefore, our findings point on the importance of developing methods able to calibrate uncertainties for each subject individually.

The results of the voxel-wise uncertainty evaluation reveal that all methods (including the softmax/sigmoid baseline) performed similarly, except for the aleatoric uncertainty, which performed worst. Among the similar performing methods, the ensemble achieved the overall best results. It achieved improved uncertainty metrics along with its expected benefits in segmentation performance. MC dropout, which can be viewed as a poor man's ensemble equivalent, showed benefits similar to ensemble when compared to using standard dropout (i.e., during training only). However, finding the optimal dropout strategy that maximizes segmentation performance and uncertainty estimates remains difficult, also because concrete dropout showed not to be optimal. Therefore, as a rule of thumb, we suggest using ensembles when resources allow it. Otherwise, we suggest applying MC dropout with a focus on regularization benefits.

The results further indicate a possible relation between the quality of the uncertainty and segmentation performance. For instance, the ensemble and the MC dropout methods revealed benefits for the uncertainty along with improved segmentation performance. It is impossible to determine whether these methods are effectively producing qualitatively better uncertainties or the increased quality results from the improved segmentation. To assess the uncertainty separately, methods that produce decoupled uncertainty estimates would be required, as advocated by Jiang et al. (2018). Our auxiliary networks are examples of such decoupled solutions. They showed promising results but without achieving substantial benefits. Further work in this direction is needed to determine its full potential. The experiment with limited training data confirmed the observation of a link between segmentation performance and quality of the uncertainties by showing improved quality with increasing dataset size. This observation is troublesome because large datasets are rare and qualitatively good uncertainties would be especially desirable for underperforming models due to little training data.

Aggregating the voxel-wise uncertainty can distill valuable information for segmentation failure detection. The best-performing aggregation method tested was the aggregation with automatically extracted features. It achieved a good correlation with the Dice coefficient and enabled an accurate separation between successful and failed segmentations results. For the mean aggregation, we obtained notably worse results, indicating a poor relation between mean uncertainty and segmentation performance. However, we could greatly improve this relation by simply weighting the uncertainties according to some prior knowledge. A subsequent feature importance analysis revealed that, particularly, the distance to the segmentation boundary matters as prior knowledge. For the aggregation with automatically extracted features, two important features in the voxel-wise uncertainty estimates were revealed: *shape sphericity* and *run length non-uniformity*. The importance of the sphericity can, to some extent, be explained by its definition, which consists of a ratio between mesh volume and surface area. This definition results in low sphericities for large-area-low-volume structures such as a narrow uncertainty rim that we would expect for a successful segmentation. Considering volume and area at the same time might be key to cope with the highly variable brain tumor volumes and areas. Similarly, the narrow uncertainty rim of successful segmentations is expected to contain a lot of similar uncertainty levels and thus resulting in a lower run length non-uniformity than of failed segmentations.

Overall, the analyzed uncertainty estimation methods only limitedly provide the desired additional and useful information. Our results question whether a remedy of the challenges with voxel-wise uncertainties is even feasible. Additional processing is required to take advantage of the voxel-wise estimates. We presented such an additional processing by aggregating the voxel-wise uncertainties into one value per subject and achieved promising results for segmentation failure detection. The promising aggregation results point in the direction of an intermediate approach, operating in-between voxel and subject level. We believe this is important in clinical applications where uncertainty estimation methods would directly operate at the levels of lesion, region, or image slice e.g., for automated segmentation correction.

Our evaluation has several limitations worth mentioning. First, due to its popularity we used a U-Net-like architecture with a shared learning scheme for all our experiments. Our findings may differ for other setups, especially when altering the output confidences of a network, such as Dice coefficient loss as shown by Sander et al. (2019). Second, the metrics used to analyze the quality are comparing with the ideal case. Although good metrics signify high quality, the opposite (i.e., bad metrics mean low quality) might not be true, since the quality is not solely defined by the employed metrics. Moreover, low metric results, as for the U-E, do not directly mean that the uncertainty information is useless but might require additional steps to create benefit. Third, we used a selection of commonly used uncertainty estimation methods. Hence, we cannot claim that these findings apply to other, recently proposed techniques (e.g., Baumgartner et al., 2019; Jena and Awate, 2019; Wang et al., 2019). Also, we analyzed the different uncertainty estimation methods independently of their expressed uncertainty (e.g., model uncertainty, data uncertainty). While this provides information on the quality across types of uncertainty, an independent analysis by type of uncertainty might bring additional insights for the development of new uncertainty estimation methods.

In conclusion, we analyzed common uncertainty estimation methods and found that the quality of their voxel-wise uncertainty is limited in terms of subject-level calibration and segmentation error localization. We further showed that aggregating the voxel-wise uncertainties to the subject level enables accurate segmentation failure detection, which after all confirms the usefulness of the uncertainty estimates. We suggest a careful usage of voxel-wise uncertainty measures and highlight the importance of developing solutions that address the subject-level requirements on calibration and segmentation error localization.

## Data Availability Statement

The datasets analyzed for this study can be found in the CBICA Image Processing Portal https://ipp.cbica.upenn.edu/.

## Author Contributions

AJ, FB, and MR contributed conception and design of the study. AJ and FB contributed implementation of the method. AJ conducted the experiments. AJ and MR wrote the manuscript. All authors contributed to manuscript revision, proofreading, and approved the submitted version.

### Conflict of Interest

The authors declare that the research was conducted in the absence of any commercial or financial relationships that could be construed as a potential conflict of interest.

## Footnotes

^1^https://pyradiomics.readthedocs.io

^2^Note that the inter-rater performance between the BraTS 2013 and the BraTS 2018 dataset might differ since the existing annotations were revised by expert board-certified neuroradiologists. Also, the non-enhancing and the necrosis label were fused.
