# Supplementary material for: Analyzing the Quality and Challenges of Uncertainty Estimations for Brain Tumor Segmentation
Source: Front Neurosci. 2020 Apr 8;14:282. doi: 10.3389/fnins.2020.00282 (PMC7156850; doi:10.3389/fnins.2020.00282)
Supplement: Supplementary file 1 [file Data_Sheet_1.pdf]

Supplementary Material

1 ARCHITECTURE DETAILS

1.1 Base Architecture and Adaptations

We used a common base architecture to analyze the different uncertainty estimation methods. Its building blocks, dimensions, and channel numbers are listed in Table S1. The 2D blocks consist of a $1\times3\times3$ convolution, dropout ($p = 0.05$), batch normalization, and ReLU activation. In the 3D blocks, the $1\times3\times3$ convolution is followed by a $3\times1\times1$ convolution, expressing the 3D convolution by a separable axial in-plane (2D) and out-plane (1D) convolution. In-plane and out-plane convolutions use *same* and *valid* border modes, respectively. Max-pooling and upsampling are performed in-plane ($1\times2\times2$) where the latter is implemented as trilinear interpolation. The input of the network is a subvolume of five consecutive axial slices of all four MR images. Only subvolumes containing information of the skull-stipped brain were used for training. If required, the subvolumes at the upper- and lowermost slices are zero-padded. The output consists of a separate probability map for each of the three hierarchical labels (WT, TC, ET) and corresponds to the subvolume's center slice.

Building block	Dimensions (Z × Y × X)	Channels
Input	5×240×240	4
2D block, 3D block, max-pool	3×120×120	32
2D block, 3D block, max-pool	1×60×60	64
2D block, 2D block, max-pool	1×30×30	128
2D block, 2D block, max-pool	1×15×15	256
2D block, 2D block	1×15×15	256
Upsample, 2D block, 2D block	1×30×30	128
Upsample, 2D block, 2D block	1×60×60	64
Upsample, 2D block, 2D block	1×120×120	32
Upsample, 2D block, 2D block	1×240×240	32
1x1 convolution	1×240×240	3
Channel-wise sigmoid	1×240×240	3

Table S1. Building blocks, dimensions, and channel numbers of the base architecture. 2D blocks consist of $1\times3\times3$ convolution, dropout ($p = 0.05$), batch normalization, and ReLU activation. 3D blocks insert an additional $3\times1\times1$ convolution after the initial convolution. Each output channel corresponds to one of the hierarchical labels (whole tumor, tumor core, and enhancing tumor).

Modifications of this base architecture are required for the different dropout strategies (*center/+MC*, *center low/+MC*, and *concrete/+MC*) and for the *aleatoric* method. The *center* strategies apply dropout directly before the max-pooling and after the upsampling instead in each 2D and 3D block. *Center/+MC* does this for all pooling/upsampling levels and *center low/+MC* only for the two lowermost levels (i.e. at dimensions $1\times60\times60$ and $1\times30\times30$). *Concrete/+MC* replaces all dropout from the base architecture by concrete dropout. The modification required for the *aleatoric* method are three additional outputs (six in total) corresponding to the variance maps of each hierarchical label. These additional outputs possess a separate 1×1 convolution layer and do not use any sigmoid classification layer. Figure S1 shows the structure of the base architecture and the modifications for the individual uncertainty estimation methods.

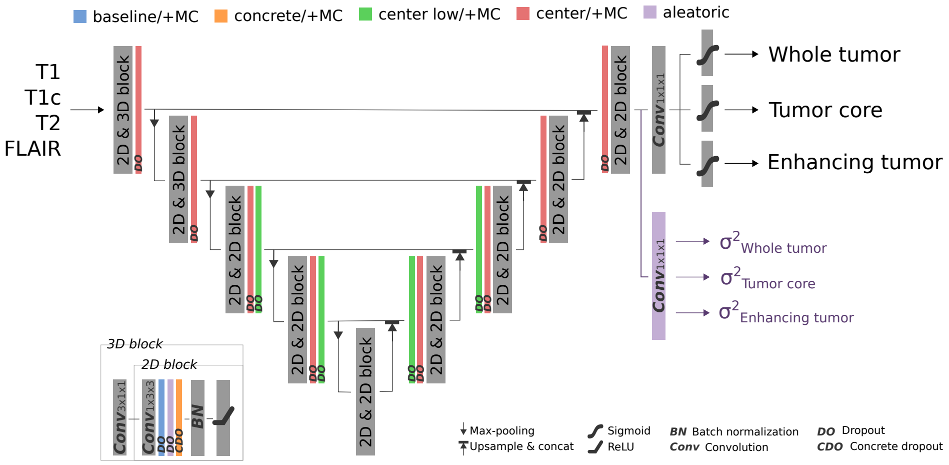

Figure S1. Structure of the base architecture including the modifications for the individual uncertainty estimation methods. The particularities for each method are color-coded.

1.2 Auxiliary Network Architectures

The *auxiliary* methods use the segmentations obtained from the base architecture (described in Section 1.1) to learn to predict the segmentation errors. To do so, *auxiliary segm.* employs a architecture equal to the base architecture but operates solely on slices (no 3D blocks) and consists of an input of seven channels. These channels correspond to the four MR images and three label maps (WT, TC, ET) produced by the base segmentation architecture. Table S2 lists the building blocks, dimensions, and channel numbers of the *auxiliary feat.* architecture. It is a lot simpler than the *auxiliary segm.* architecture since it employs direct feature information, in contrast to the label maps, of the base segmentation network. It consists of three consecutive 2D blocks, each comprising a 1×1 convolution, batch normalization, and ReLU activation. Figure S2 shows the structure of both auxiliary architectures.

Besides possessing different architectures, the two auxiliary methods also differ in the computation of their training data. While *auxiliary feat.* was trained on the errors that the segmentation network made on the training data, *auxiliary segm.* used the segmented label maps of a five-fold cross-validation of the training set. This cross-validation ensures that *auxiliary segm.* is trained on errors produced on held out (test) splits of the training data rather than training set errors.

As for the base architecture, we used Adam optimizer (learning rate: 10^{-4}, β_1: 0.9, β_2: 0.999, ε: 10^{-8}) to optimize the cross-entropy loss in mini-batches of 24.

Building block	Dimensions (Y × X)	Channels
Input	240×240	32
2D block	240×240	32
2D block	240×240	32
2D block	240×240	32
1x1 convolution	240×240	3
Channel-wise sigmoid	240×240	3

Table S2. Building blocks, dimensions, and channel numbers of the architecture used for *auxiliary feat.* method. 2D blocks consist of $1\times3\times3$ convolution, batch normalization, and ReLU activation. Each output channel corresponds to the uncertainty of one of the hierarchical labels (whole tumor, tumor core, and enhancing tumor).

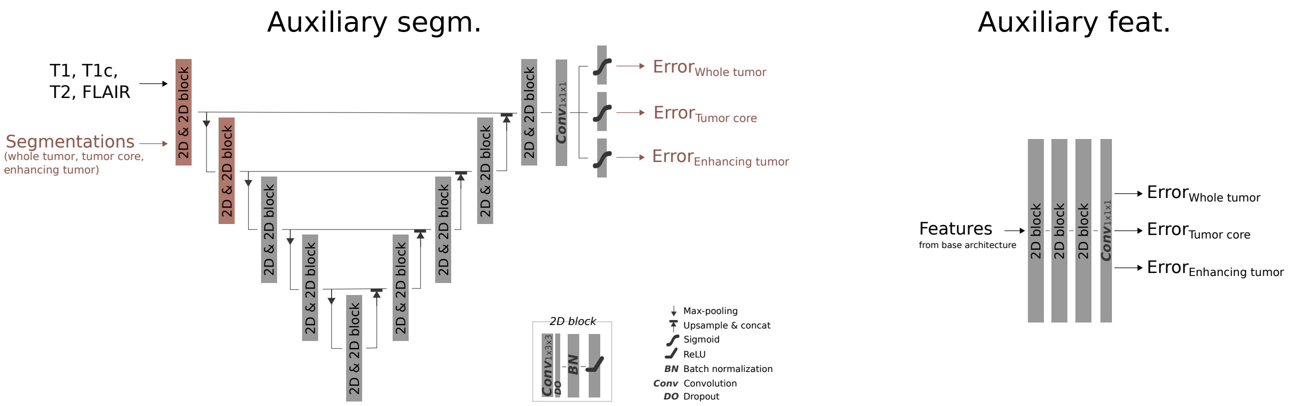

Figure S2. Structure of the *auxiliary segm.* and *auxiliary feat.* architectures. The modifications of the *auxiliary segm.* architecture compared to the base architecture are shown in brown.

2 AGGREGATION METHODS

2.1 Prior Knowledge Aggregation

We used three types of voxel-wise uncertainty weighting during aggregation with prior knowledge: (a) boundary weighting, (b) distance weighting, and (c) volume weighting. Boundary weighing excludes uncertainties at the segmentation boundary, which we defined as a three-voxel band (i.e., boundary and voxels next to the boundary). Distance weighting weights the uncertainties by the Euclidean distance to the segmentation boundary in voxels (i.e., distance increases by one for each voxel). Volume weighting inversely weights the overall uncertainty by the volume of the segmentation. We combined these three weighting methods with three simple aggregation operations: mean, sum, and logsum aggregation. Table S3 lists the nine combinations we considered.

Type	Description
mean	Average of the voxel-wise uncertainties of a subject
logsum	Sum of the voxel-wise log uncertainties of a subject
volume weighted sum	Sum of the voxel-wise uncertainties divided by the segmentation volume
masked mean	Mean of the uncertainties masked by the segmentation boundary
masked logsum	Logsum of the uncertainties masked by the segmentation boundary
dist. w. mean	Mean of the distance weighted uncertainties
dist. w. logsum	Logsum of the distance weighted uncertainties
dist. w. masked mean	Mean of the distance weighted and boundary masked uncertainties
dist. w. masked logsum	Logsum of the distance weighted and boundary masked uncertainties

Table S3. List of the nine different uncertainty aggregations with prior knowledge.

2.2 Automatically Extracted Features

As mentioned in the main text, we used `PyRadiomics` (version 2.2.0) to automatically extract features from the voxel-wise uncertainty estimates. We defined the region of interest by thresholding the uncertainty with the thresholds that achieved the best validation set performance for the U-E metric (same thresholds as used to calculate the U-E metric). We extracted the 102 default `PyRadiomics` features consisting of 14 shape features, 18 first-order statistic features, 24 gray level[1] co-occurrence matrix features, 16 gray level run length matrix features, 16 gray level size zone matrix features, and 14 gray level dependence matrix features. The default settings were used for the extraction (image type: original, bin width: 25). For details on the features groups and individual features we refer to the `PyRadiomics` website[2].

2.3 Random Forest Regressor

To predict the Dice coefficient of the segmentations from the uncertainty features (aggregated by prior knowledge or automatically extracted), we used the random forest regressor from `scikit-learn`[3] (version 0.21.2) with the default settings (n_estimators: 10, criterion: 'mse' max_depth: None). To train the random forest, we applied a five-fold cross-validation on the 160 subjects of the test set and repeated this process five times. The final prediction and features importances were obtained by averaging the results of the five repetitions. Note that we purposely did not tune any parameters in this cross-validation.

3 VISUAL EXAMPLES OF UNCERTAINTY ESTIMATES

Figure S3 and S4 show the uncertainty estimates for the tumor core and enhancing tumor labels produced by the selected methods on underconfident, overconfident, and well-calibrated subjects.

4 ADDITIONAL VOXEL-WISE METRICS

We computed additional metrics that complement the expected calibration error (ECE) and the uncertainty-error overlap (U-E) from the main text.

ACE. The average calibration error (ACE) aims at distilling the information of a reliability diagram into one scalar value. In contrast to the ECE, the absolute calibration error between the confidence and accuracy bins, c_m and a_m respectively, is equally weighted in the ACE. With M being the number of non-empty bins, the ACE is given by

$$ACE = \frac{1}{M} \sum_{m}^{M} |c_m - a_m| \, ,$$

and ranges from 0 to 1, where a lower value represents a better calibration. As for the ECE, we report the mean subject ACE.

AUC-PR. Complementary to the uncertainty-error overlap, we also report the area under the curve of the precision-recall curve (AUC-PR). The AUC-PR summarizes the precision and recall performance of the overlap between uncertainty and segmentation error at different thresholds. We used the same thresholds as for determining the best U-E. The AUC-PR ranges from 0 to 1, where higher AUC-PR values indicate better performance.

[1] In our case the gray levels are the uncertainty levels

[2] https://pyradiomics.readthedocs.io

[3] https://scikit-learn.org/stable/index.html

	WT		TC		ET	
	ACE%	AUC-PR	ACE%	AUC-PR	ACE%	AUC-PR
baseline	16.995	0.249	17.905	0.243	19.951	0.23
concrete	15.65	0.251	17.77	0.253	19.118	0.229
center low	15.569	0.251	17.667	0.25	19.493	0.234
center	19.886	0.238	18.998	0.247	19.532	0.22
baseline + MC	16.505	0.248	16.997	0.24	18.598	0.221
concrete + MC	15.571	0.249	17.554	0.251	18.759	0.227
center low + MC	15.346	0.244	17.523	0.248	19.076	0.231
center + MC	**13.553**	0.205	17.257	0.213	17.827	0.19
ensemble	15.702	0.24	16.719	0.23	18.164	0.219
aleatoric	15.518	0.004	**10.412**	0.202	**14.51**	0.029
auxiliary segm.	17.677	0.298	20.252	0.249	21.416	0.284
auxiliary feat.	17.278	**0.301**	17.918	**0.276**	21.033	**0.344**

Table S4. Performances of the different uncertainty estimation methods in terms of average calibration error (ACE) and area under the curve of the precision-recall curve (AUC-PR). Both metrics range from 0 to 1, but the ACE is reported in %. Lower ACE values are better as well as higher AUC-PR values. Bold values indicate best performances. Horizontal separations group types of uncertainty methods and WT, TC, and ET indicate the tumor regions whole tumor, tumor core, and enhancing tumor.

Table S4 lists the results obtained for the ACE and AUC-PR metrics. The main differences of the ACE results compared to the ECE results (see Table 1 in the main text) are the good ACE results obtained by the *aleatoric* method. This is mainly due to the empty bins, which are ignored for the ACE. We also observe good ACE results for the *center+MC* method. We expect this to be due to a more equal distribution of the samples among the bins compared to the other methods. In terms of AUC-PR we observe a benefit of the *auxiliary* methods. The reason might be related to their training, in which are optimized to find the overlap between the automated segmentation and the reference segmentation. Also, the *+MC* versions consistently perform worse than their counterparts.

5 DETAILS ON AGGREGATION RESULTS

5.1 ROC Curves

Figure S5 shows the receiver operating characteristic (ROC) curves of the uncertainty estimation methods for the three aggregation methods and three tumor regions.

5.2 Metric Values

Table S5 presents the details of the obtained failure detection (AUC-ROC, Youden's accuracy) and the correlation to the Dice coefficient (Spearman's rank correlation) for the three aggregation methods: (a) mean aggregation, (b) aggregation with prior knowledge, and (c) aggregation with automatically extracted features.

		AUC-ROC			ρ			Youden		
		WT	TC	ET	WT	TC	ET	WT	TC	ET
**Mean**	baseline	0.443	0.575	0.336	-0.067	0.145	-0.220	0.712	0.700	0.675
	concrete	0.619	0.568	0.244	0.126	0.193	-0.407	0.781	0.725	0.656
	center low	0.565	0.570	0.241	0.073	0.213	-0.424	0.781	0.738	0.350
	center	0.450	0.516	0.366	-0.128	-0.030	-0.220	0.588	0.644	0.425
	baseline + MC	0.483	0.562	0.338	0.001	0.146	-0.232	0.738	0.712	0.669
	concrete + MC	0.598	0.580	0.242	0.155	0.226	-0.398	0.788	0.625	0.681
	center low + MC	0.612	0.570	0.252	0.131	0.220	-0.400	0.712	0.731	0.350
	center + MC	0.438	0.497	0.434	0.010	0.004	-0.176	0.725	0.644	0.594
	ensemble	0.498	0.607	0.318	0.070	0.196	-0.210	0.756	0.562	0.706
	aleatoric	0.413	0.655	0.501	-0.177	0.266	-0.134	0.312	0.675	0.444
	auxiliary segm.	0.475	0.601	0.229	-0.009	0.183	-0.404	0.725	0.694	0.331
	auxiliary feat.	0.464	0.591	0.267	-0.035	0.175	-0.334	0.694	0.706	0.338
**Prior knowledge**	baseline	0.734	0.817	0.811	0.438	0.660	0.550	0.706	0.744	0.794
	concrete	0.794	0.841	0.833	0.540	0.631	0.496	0.675	0.731	0.762
	center low	0.754	0.821	0.852	0.479	0.612	0.635	0.700	0.794	0.756
	center	0.708	0.754	0.776	0.351	0.469	0.514	0.656	0.738	0.719
	baseline + MC	0.749	0.832	0.762	0.510	0.631	0.462	0.644	0.756	0.688
	concrete + MC	0.818	0.811	0.818	0.549	0.579	0.550	0.688	0.700	0.775
	center low + MC	0.777	0.801	0.829	0.489	0.611	0.606	0.738	0.769	0.750
	center + MC	0.719	0.689	0.792	0.333	0.381	0.565	0.594	0.644	0.731
	ensemble	0.770	0.858	0.822	0.500	0.666	0.480	0.712	0.794	0.800
	aleatoric	0.556	0.642	0.649	0.185	0.284	0.301	0.700	0.662	0.669
	auxiliary segm.	0.812	0.822	0.804	0.633	0.648	0.510	0.712	0.731	0.762
	auxiliary feat.	0.859	0.840	0.817	0.619	0.714	0.525	0.756	0.756	0.738
**Auto. extracted features**	baseline	0.928	0.939	0.895	0.771	0.882	0.701	0.888	0.894	0.800
	concrete	0.879	0.946	0.911	0.752	0.882	0.732	0.831	0.856	0.875
	center low	0.892	0.940	0.920	0.716	0.867	0.748	0.856	0.888	0.856
	center	0.893	0.899	0.843	0.737	0.784	0.656	0.869	0.812	0.812
	baseline + MC	0.934	0.954	0.890	0.791	0.887	0.706	0.888	0.856	0.838
	concrete + MC	0.912	0.956	0.924	0.765	0.893	0.752	0.875	0.819	0.869
	center low + MC	0.902	0.946	0.920	0.748	0.883	0.744	0.825	0.912	0.844
	center + MC	0.891	0.879	0.869	0.731	0.792	0.717	0.881	0.769	0.806
	ensemble	0.919	0.961	0.893	0.765	0.883	0.640	0.806	0.919	0.819
	aleatoric	0.586	0.373	0.719	0.260	-0.174	0.454	0.531	0.612	0.750
	auxiliary segm.	0.911	0.909	0.880	0.817	0.835	0.704	0.856	0.838	0.825
	auxiliary feat.	0.907	0.941	0.871	0.725	0.884	0.690	0.862	0.831	0.731

Table S5. Aggregation results obtained by the three aggregation methods: mean aggregation, aggregation with prior knowledge, and aggregation with automatically extracted features. The area under the curve of the receiver operating characteristic (AUC-ROC) and Youden's accuracy indicate the goodness of failure detection and Spearman's rank (ρ) shows the correlations to the achieved Dice coefficient. WT, TC, and ET indicate the tumor regions whole tumor, tumor core, and enhancing tumor.

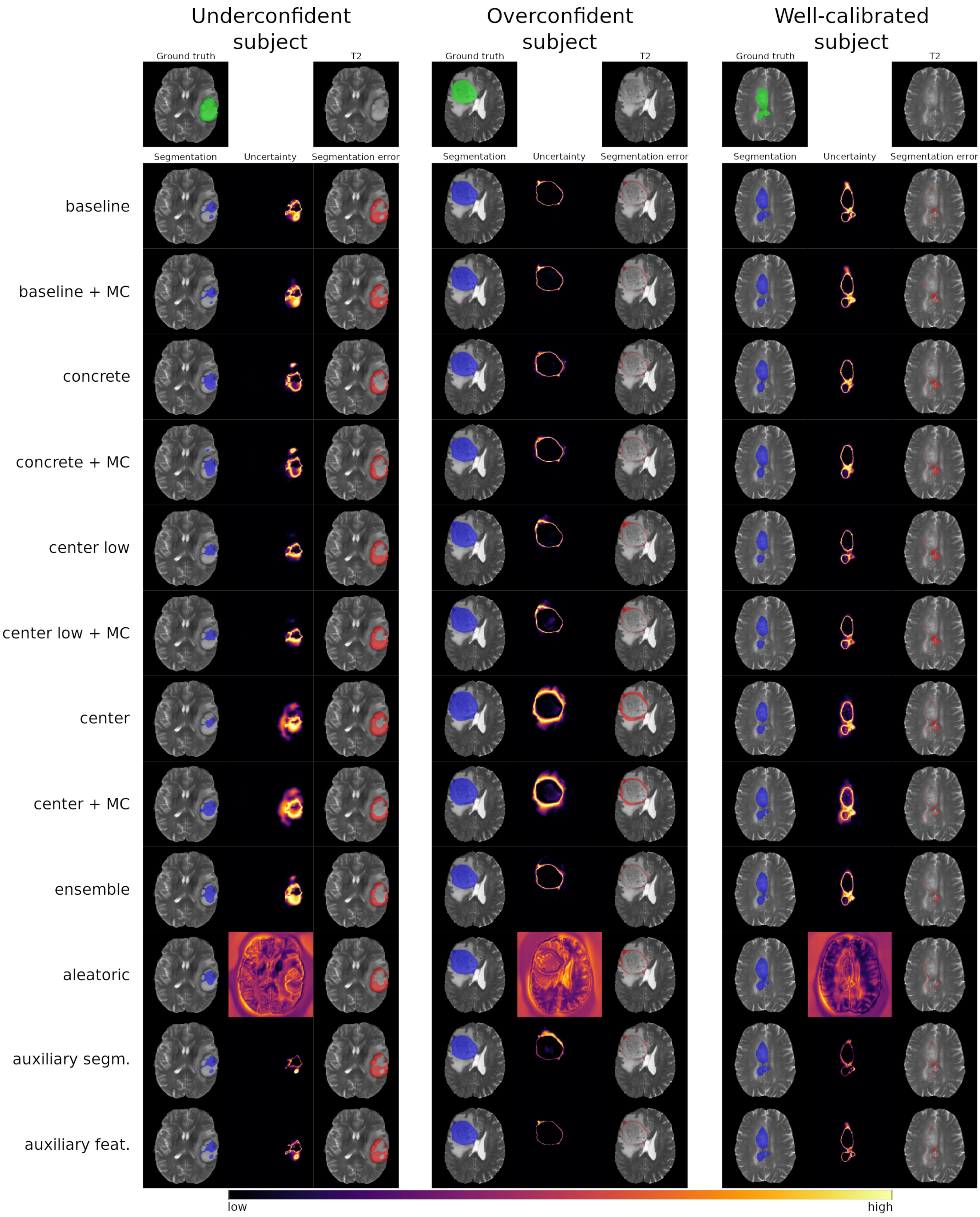

Figure S3. Visual examples of the tumor core uncertainty produced by the different uncertainty estimation methods. The columns correspond to underconfident, overconfident, and well-calibrated subjects.

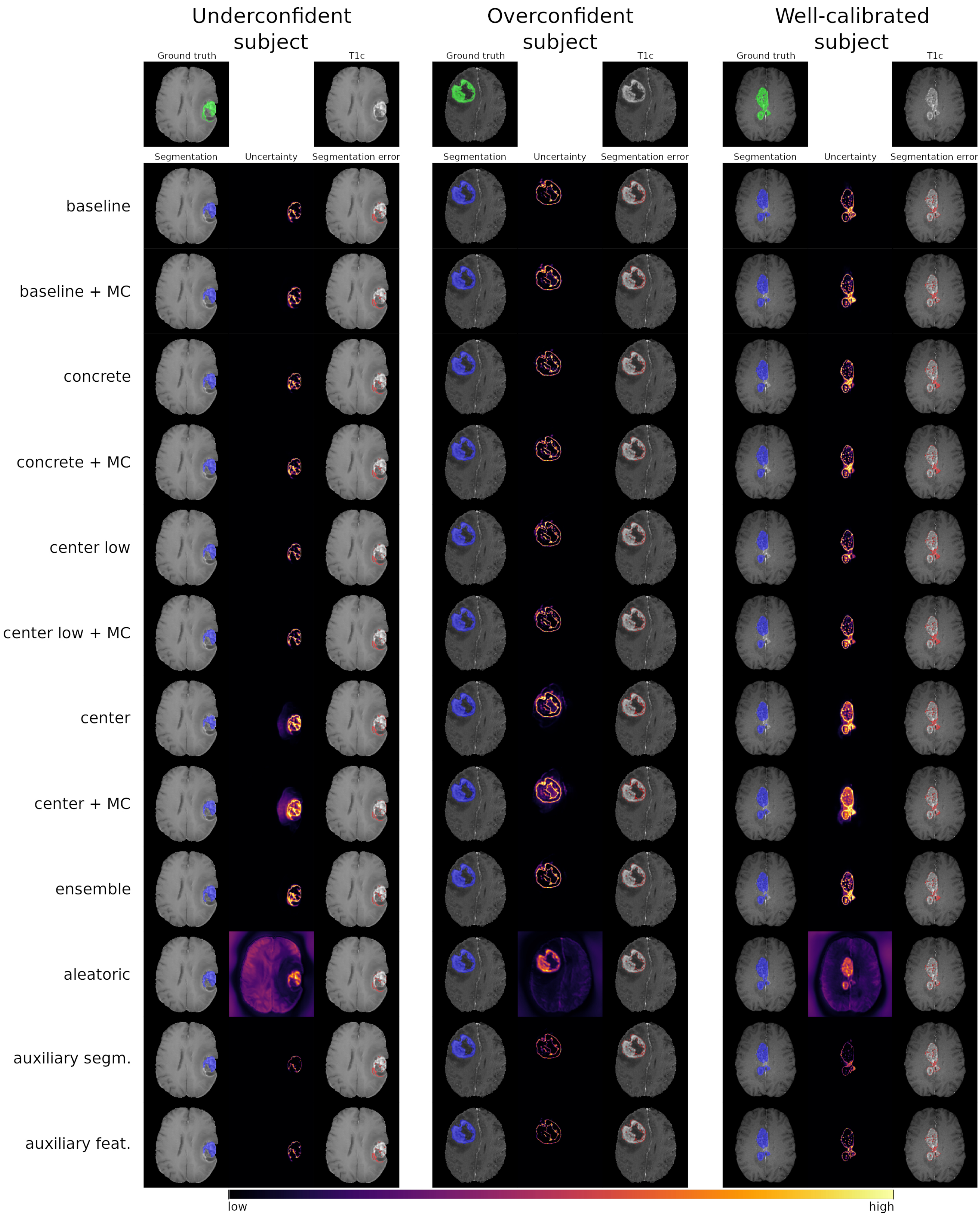

Figure S4. Visual examples of the enhancing tumor uncertainty produced by the different uncertainty estimation methods. The columns correspond to underconfident, overconfident, and well-calibrated subjects.

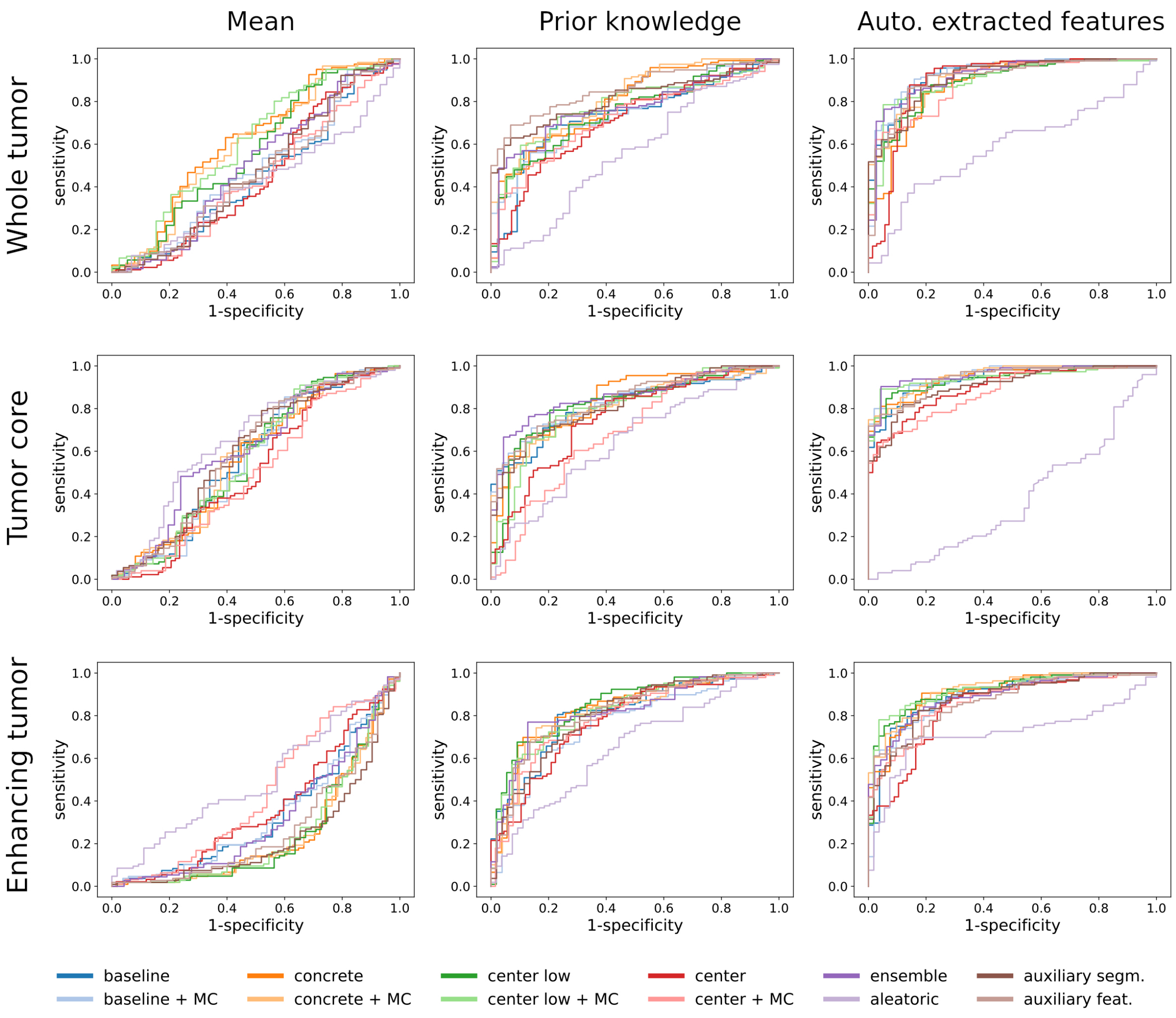

Figure S5. Receiver operating characteristic (ROC) curves of the selected uncertainty estimation methods. The columns represent the aggregation methods: mean aggregation, aggregation with prior knowledge, and aggregation with automatically extracted features. The rows indicate the three tumor regions.
